# Comments on: risk factors for nosocomial meningitis in patients with external ventricular drainages

**DOI:** 10.1186/s40560-026-00851-0

**Published:** 2026-01-26

**Authors:** Maxime Théo Aparicio, Sylvain Diop, Roman Mounier, SisyPh Research Group, Roman Mounier, Sylvain Diop, Maxime Aparicio, Ariane Roujansky, Hatem Kallel

**Affiliations:** 1 Department of Anesthesiology and Critical Care, Georges Pompidou European Hospital, Assistance Publique-Hôpitaux de Paris, 20 Rue Leblanc, 75015 Paris, France; 2 Department of Anesthesiology, Marie Lannelongue Hospital, Le Plessis Robinson, France; 3 Cardiothoracic Intensive Care Unit, Marie Lannelongue Hospital, Le Plessis Robinson, France; 4 Department of Anaesthesiology and Critical Care, Avicenne Hospital, Assistance Publique-Hôpitaux de Paris, France University, Sorbonne Paris Nord, Bobigny, France; 5 Université Sorbonne Paris Nord, Villetaneuse, France

## Abstract

In this comment, we aim to offer some perspective on the work of Raffenot *et al.* entitled “Risk factors for nosocomial meningitis in patients with external ventricular drainages”. After highlighting the main findings of their study, we discuss the predictive model as well as the disease definition they used.

To the editor,

We read with great interest the work of Raffenot *et al.* recently published in the Journal of Intensive Care [1]. In this article, the authors retrospectively explore risk factors of nosocomial meningitis (*i.e*., external ventricular drain-associated infections) among patients undergoing external cerebrospinal fluid (CSF) drainage. They have been able to design a model including β-lactam allergy, CSF leakage, prolonged drainage, and cerebrospinal biological profile for predicting those device-related infections with appreciable performance (Receiver Operating Characteristic_Area Under Curve_ of 0.89, a specificity of 88.3%, a sensitivity of 84.2% and a negative predictive value of 96.8%).

Despite an extensive exploration of nosocomial meningitis risk factors over the last five decades, Raffenot *et al.* are the first ones to propose that β-lactam allergy might be considered as a risk factor for nosocomial meningitis onset—indirectly, as underlined by authors themselves. They suggest that allergic patients might be more inclined to those infections consecutively to the eviction of β-lactam that are otherwise widely used among critically ill patients as first line antimicrobial therapy for most of the infections they undergo. This finding is of major importance—despite the administration occurring below meningeal doses—since it allows to consider the patient as a whole, and might be a founding piece of evidence for “inter-infection interactions” consideration in future research.

With an overall 11.6% incidental rate of nosocomial meningitis despite quite prolonged median drainage durations (15 and 19 days for those without and with infection, respectively), their results are within the range literature suggests [2]. This result suggests that their model might help discriminate earlier infected ones from the others.

Nosocomial meningitis predictive models then need to reach “excellent discrimination” as underlined by authors themselves. Indeed, the main challenge of nosocomial meningitis diagnosis—a rare event among critically ill patients prone to any kind of infection—is its accuracy. In such cohort designs, a very high negative predictive value as reported by Raffenot *et al.* is expected, as a mechanical consequence of cohort’s characteristics. However, the clinical problem is to decide whether our patients *have developed* an infection, so to focus on the positive predictive value might be more “real-life” accurate.

Considering the characteristics authors reported (sensitivity of 84.2%, specificity of 88.3%, and prevalence of 11.6%), their model reaches a positive predictive value of 48.6%. In other words, among all patients that are predicted “infected” by the model, less than a half of them actually complies with the definition criteria they selected for nosocomial meningitis (*i.e*., CSF leukocyte count > 100 cells/mm^3^, CSF protein > 0.4 g/L, CSF-to-serum glucose ratio < 0.5 associated with or without a bacteriological confirmation since authors gathered Lozier’s suspected and proven ventriculostomy-related infections and ventriculitis [3]).

The recommended empirical antimicrobials for nosocomial meningitis treatment are wide spectrum hydrophilic drugs administered at high dosage in order to reach appropriate concentrations in the nervous side of the blood–brain barrier. Such therapeutics expose patients to both usual antibiotic-related bacteriological pitfalls such as resistance emergence, but since those patients are often dealing with several organ failures, such dosages expose them to serious toxicity concerns. To us, this emphasizes the need for cautious use of such models, especially when used as monitoring parameters, since they could encourage preemptive administration of unnecessary broad-spectrum antimicrobial therapies for a significant proportion of patients.

Furthermore, the very definition criteria of nosocomial meningitis among externally derived patients is not trivial [4]. Indeed, the authors, rather than retrospectively considering the patients they treated for nosocomial meningitis among their cohort, used criteria inspired by Lozier *et al*.[3], but did not encompass those proposed by the CDC/IDSA guidelines [5]. Among others, the main differences in Lozier’s criteria compared to CDC/IDSA’s are that clinical signs without any better explanation could not define by themself a nosocomial meningitis, and neither could a cultured organism from the CSF by itself. So, if CDC/IDSA guidelines allow clinicians to define an aseptic nosocomial meningitis, this entity would only be considered as “suspected ventriculostomy-related infection” according to Lozier’s criteria. Conversely, the isolation of microorganisms cultured from CSF—a frequent issue when dealing with percutaneous devices—while sufficient to define CDC/IDSA nosocomial meningitis would not by itself meet Lozier’s “ventriculostomy-related infection” definition. These differences are a major issue in the understanding of device-related neurosurgical infections, as they do not encompass the same clinical contexts, and since predictive models, as discussed before, are widely influenced by the actual prevalence in the considered cohorts.

If authors chose a pragmatic definition that they were able to properly analyze retrospectively, then neglecting the clinical consequences of the infection (that Lozier *et al.* used to distinguish ventriculostomy-related infections and ventriculitis), it should be emphasized that this definitional question is crucial, since actual clinical performance of predictive models depends on the actual incidental rate of the cohort considered. Indeed, the positive predictive value of 48.6% reported by the authors is largely a consequence of the low prevalence of nosocomial meningitis in their cohort.

Finally, it seems important to underline that since the authors encompass biochemical CSF modifications in both risk factors (predictors) and infection definition (outcome) in the tested model, despite slightly different threshold (CSF-to-serum glucose ratio < 0.5 with CSF protein > 1 g/L as a risk factor; and CSF-to-serum glucose ratio < 0.5 with CSF protein > 0.4 g/L as a constitutive part of their outcome), the interpretation of the model’s reliability must be prudent. Indeed, such model design induces verification bias (partial incorporation bias in the present case) and circularity, potentially inflating prevalence-independent performance metrics (*e.g*., sensitivity and specificity). Prospective external validation in appropriately selected patients in order to enhance pre-test probability is needed to properly evaluate its performance.

## Data Availability

No datasets were generated or analysed during the current study.

## References

[1] Raffenot C, Doat-Sarfati V, Antonini F, Florant T, Baron S, Agard G, et al. Risk factors for nosocomial meningitis in patients with external ventricular drainages. J Intensive Care. 2025;13(1):63. 10.1186/s40560-025-00828-5. 41219997 10.1186/s40560-025-00828-5 PMC12604254

[2] Ramanan M, Lipman J, Shorr A, Shankar A. A meta-analysis of ventriculostomy-associated cerebrospinal fluid infections. BMC Infect Dis. 2015;15(1):3. 10.1186/s12879-014-0712-z. 25567583 10.1186/s12879-014-0712-z PMC4300210

[3] Lozier AP, Sciacca RR, Romagnoli MF, Connolly ES. Ventriculostomy-related infections: a critical review of the literature. Neurosurgery. 2002;51:170–82. 10.1097/00006123-200207000-00024. 12182415 10.1097/00006123-200207000-00024

[4] Karvouniaris M, Brotis A, Tsiakos K, Palli E, Koulenti D. Current perspectives on the diagnosis and management of healthcare-associated ventriculitis and meningitis. Infect Drug Resist. 2022;15:697–721. 10.2147/IDR.S326456. 35250284 10.2147/IDR.S326456 PMC8896765

[5] Tunkel AR, Hasbun R, Bhimraj A, Byers K, Kaplan SL, Scheld WM, et al. 2017 infectious diseases society of america’s clinical practice guidelines for healthcare-associated ventriculitis and meningitis. Clin Infect Dis. 2017;64:e34-65. 10.1093/cid/ciw861. 28203777 10.1093/cid/ciw861 PMC5848239

